# Molecular identification of four *Sarcocystis* species in the herring gull, *Larus argentatus*, from Lithuania

**DOI:** 10.1186/s13071-019-3869-x

**Published:** 2020-01-06

**Authors:** Petras Prakas, Dalius Butkauskas, Evelina Juozaitytė-Ngugu

**Affiliations:** Nature Research Centre, Akademijos Str. 2, Vilnius, 08412 Lithuania

**Keywords:** *Sarcocystis*, Herring gull, ITS1, Species differentiation

## Abstract

**Background:**

Birds of the family Laridae have not been intensively examined for infections with *Sarcocystis* spp. To date, sarcocysts of two species, *S*. *lari* and *S*. *wobeseri*, have been identified in the muscles of gulls. The aim of the present study was to evaluate the species richness of *Sarcocystis* in the herring gull, *Larus argentatus*, from Lithuania.

**Methods:**

In the period between 2013 and 2019, leg muscles of 35 herring gulls were examined for sarcocysts of *Sarcocystis* spp. *Sarcocystis* spp. were characterised morphologically based on a light microscopy study. Four sarcocysts isolated from the muscles of each infected bird were subjected to further molecular examination. *Sarcocystis* species were identified by means of ITS1 sequence analysis.

**Results:**

Sarcocysts were detected in 9/35 herring gulls (25.7%). Using light microscopy, one morphological type of sarcocysts was observed. Sarcocysts were microscopic, thread-like, had a smooth and thin (about 1 µm) cyst wall and were filled with banana-shaped bradyzoites. On the basis of ITS1 sequences, four *Sarcocystis* species, *S*. *columbae*, *S*. *halieti*, *S*. *lari* and *S*. *wobeseri*, were identified. Furthermore, it was demonstrated that a single infected herring gull could host two *Sarcocystis* species indistinguishable under light microscopy.

**Conclusions:**

*Larus argentatus* is the first bird species found to act as intermediate host of four *Sarcocystis* spp. According to current knowledge, five species, *S*. *falcatula*, *S*. *calchasi*, *S*. *wobeseri*, *S*. *columbae* and *S*. *halieti* can use birds belonging to different orders as intermediate hosts. 
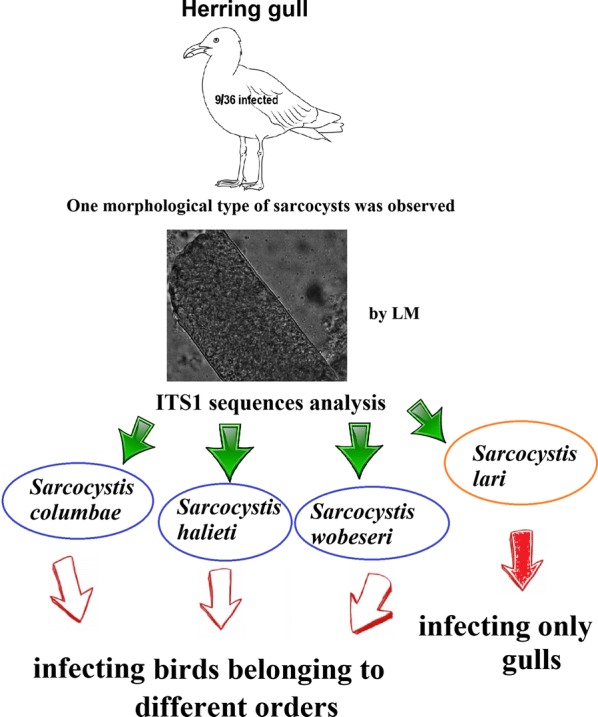

## Background

Protozoan parasites of the genus *Sarcocystis* are cyst-forming coccidians having an obligatory two-host prey-predator life-cycle [1, 2]. Asexual multiplication occurs in the intermediate host (IH), whereas sexual multiplication takes place in the small intestine of the definitive host (DH) [2, 3]. Thus far over 200 *Sarcocystis* species have been described; however, a much higher number or species diversity of these parasites is presumed [1, 4].

Birds may serve as intermediate or definitive hosts for many *Sarcocystis* species [1, 5–8]. More than 25 *Sarcocystis* species have been identified using birds as intermediate hosts [1, 9]. Two species, *S*. *falcatula* and *S*. *calchasi* are highly pathogenic for their intermediate hosts. Some species, such as *S*. *falcatula*, *S*. *calchasi* and *S*. *wobeseri* are not strictly specific to the intermediate host and could form sarcocysts in birds of several different orders [10–13]. By contrast, other species like *S*. *fulicae*, *S*. *lari* and *S*. *ramphastosi* are strictly specific to a single bird species [14–16].

Herring gulls are opportunistic predators of marine invertebrates, fishes, insects and birds, as well as opportunistic scavengers of dead animals and garbage [17, 18]. To date, only two *Sarcocystis* species, *S*. *lari* and *S*. *wobeseri*, have been described in birds of the family Laridae [15, 19]. The present study provides molecular identification of four *Sarcocystis* species from *L*. *argentatus* that are morphologically indistinguishable under light microscopy examination.

## Methods

### Collection of samples

A total of 35 herring gulls, road-killed and received from taxidermists between 2013 and 2019 were studied. Leg muscles were examined for the presence of sarcocysts.

### Morphological analysis

The prevalence and intensity of infection with *Sarcocystis* spp. was evaluated in methylene blue-stained preparations. For this purpose, 28 oat-size fragments (about 1 g) of muscles were cut-off, stained with 0.2% methylene blue solution, clarified with 1.5% acetic acid solution and pressed in a glass compressor. After squeezing of fresh muscle tissues, sarcocysts were excised with the help of preparation needles and then morphologically characterized under a light microscope (LM).

### DNA extraction and PCR

Four sarcocysts were extracted from the leg muscles of each infected bird and subjected to light microscopy and molecular investigation. For the molecular analysis, sarcocysts were placed in individual 1.5 ml tubes containing 20 μl of 96% ethanol and kept at − 20 °C. Genomic DNA was extracted from individual sarcocysts using the GeneJET Genomic DNA Purification Kit (Thermo Fisher Scientific Baltics, Vilnius, Lithuania) according to the manufacturer’s recommendations.

The complete ITS1 region was amplified using the SU1F/5.8SR2 primer pair [20]. Each PCR mixture consisted of 25 μl, containing 12.5 μl of Dream-Taq PCR Master Mix (Thermo Fisher Scientific, Waltham, US), 0.5 μM of each primer, 0.02 μg template DNA and nuclease-free water. The cycling conditions began with one cycle at 95 °C for 5 min followed by 35 cycles of 94 °C for 45 s, 60 °C for 60 s and 72 °C for 80 s, and a final extension step at 72 °C for 7 min. PCR products were evaluated using a 1.5% agarose gel and visualized *via* UV light after staining with 0.05 µg/ml ethidium bromide. Amplified DNA fragments were purified with exonuclease *Exo*I and alkaline phosphatase FastAP (Thermo Fisher Scientific).

### DNA sequencing, sequence alignment and phylogenetic analysis

Sequencing reactions were performed using the Big-Dye Terminator v3.1 Cycle Sequencing Kit and the 3500 Genetic Analyzer (Applied Biosystems, Foster City, California, USA) according to the manufacturer’s recommendations. PCR products were sequenced directly using the PCR forward and reverse primers. The ITS1 sequences obtained in this study were compared with those of various *Sarcocystis* spp. using the Nucleotide BLAST program (megablast option). Sequences were aligned using the MUSCLE algorithm implemented in MEGA7 [21] software. The TOPALi v2.5 software [22] was used to select a nucleotide substitution model with the best fit to the aligned sequence dataset and to construct the phylogenetic tree under the Bayesian inference. Sequences for *Sarcocystis* spp. from *L*. *argentatus* generated in the present study are deposited in the GenBank database under the accession numbers MN450338-MN450373.

## Results

Sarcocysts were detected in 9 out of 35 (25.7%) herring gulls examined in Lithuania. The infection intensity of *Sarcocystis* spp. sarcocysts in 1 g of the leg muscle in *L*. *argentatus* varied from 1 to 85 cysts (mean = 33.0, median = 19.0). Examination of 36 sarcocysts under LM revelaed that they are morphologically similar. Sarcocysts were microscopic, thread-like, 2860–7930 × 43–200 μm in size, with a thin (0.7–1.5 μm), apparently smooth cyst wall. Septa divided sarcocysts into compartments filled with banana-shaped bradyzoites, 5.5–9.0 × 1.2–2.4 μm in size.

**Fig. 1:**
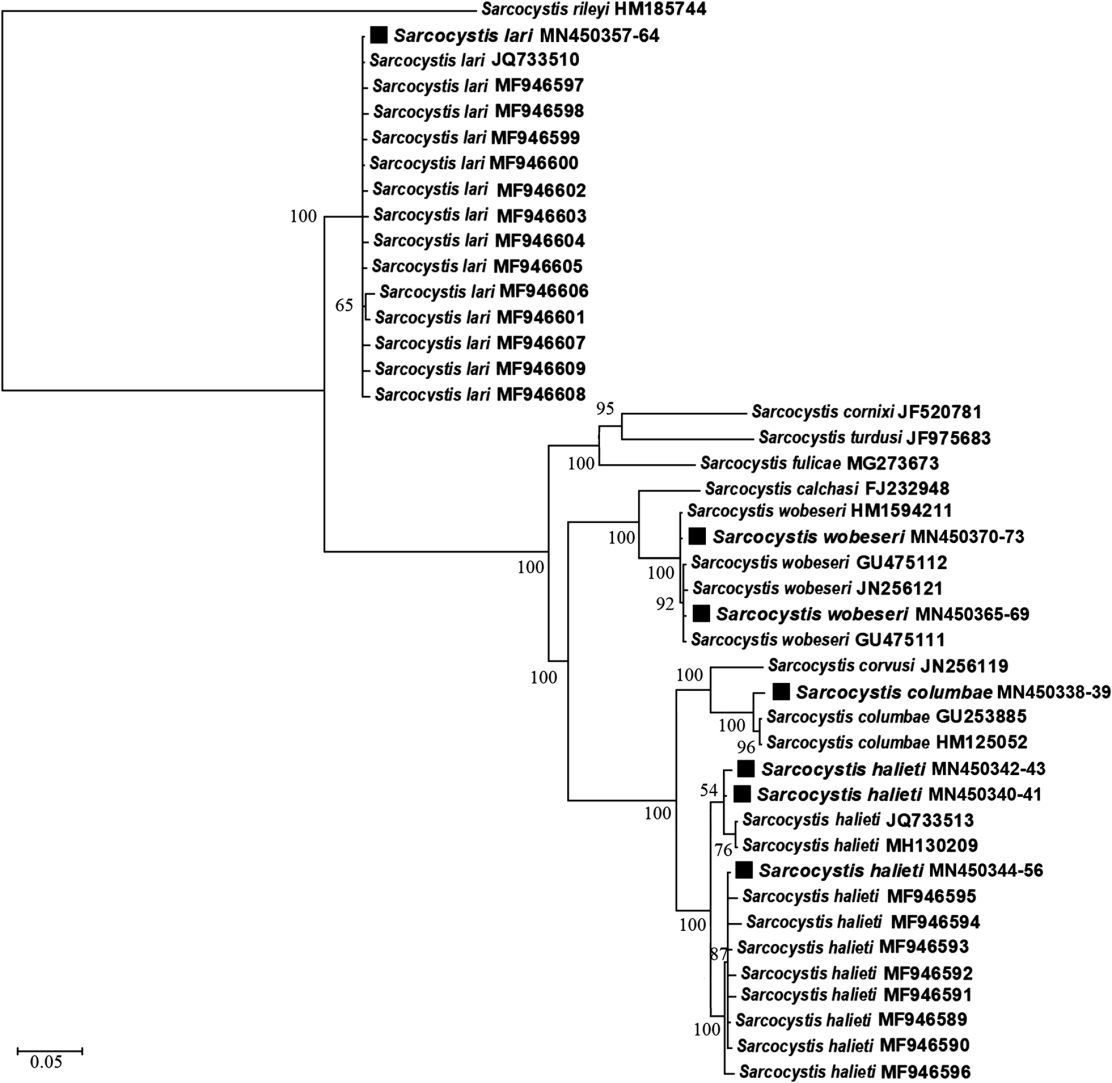
Phylogenetic tree of selected *Sarcocystis* species based on ITS1 sequences. The figures next to branches show the posterior probability support values. Sequences generated in the present study are indicated with squares

Surprisingly, the comparison of ITS1 sequences showed that the morphologically similar sarcocysts belonged to four different species of *Sarcocystis*, *S*. *columbae*, *S*. *halieti*, *S*. *lari* and *S*. *wobeseri* (Fig. 1). In the phylogenetic tree, the examined *Sarcocystis* spp. were placed into single-species clusters with a maximum support value. Based on ITS1, 833-bp long sequences of *S*. *columbae* obtained from *L*. *argentatus* (GenBank: MN450338-MN450339) demonstrated 99.9–100% identity with those of *S. columbae* (GenBank: GU253885, HM125052) from the wood pigeon (*Columba palumbus*). The BLAST analysis revealed that 860-bp long sequences of *S*. *lari* from *L*. *argentatus* (MN450357-MN450364) shared 99.1–100% identity with those of *S*. *lari* from the black-backed gull (*Larus marinus*) (IH, GenBank: JQ733510) and from the white-tailed sea eagle (*Haliaeetus albicilla*) (DH, GenBank: MF946597-MF946609). The 844-bp long ITS1 sequences of *S*. *wobeseri* obtained in this study (MN450365-MN450373) showed 99.8–100% identity with other sequences of *S*. *wobeseri* from the mallard duck (*Anas platyrhynchos*) (GenBank: JN256121), the barnacle goose (*Branta leucopsis*) (GenBank: GU475111) and *L*. *argentatus* (GenBank: HM159421). At ITS1, 830-bp long sequences of *S*. *halieti* from *L*. *argentatus* (MN450340-MN450356) shared 98.1–100% identity with other sequences of *S*. *halieti* from the great cormorant (*Phalacrocorax carbo*) (IH; GenBank: MH130209, JQ733513) and *H*. *albicilla* (DH; GenBank: MF946589-MF946596).

Two *Sarcocystis* species identified in the present study, *S*. *columbae* (*n *= 2) and *S*. *lari* (*n *= 8) did not show any intraspecific genetic variability. The obtained ITS1 sequences of *S*. *wobeseri* differed only by one SNP (A/G) at nucleotide position 120, whereas *S*. *halieti* sequences demonstrated 98.7–100% identity. Thirteen identical sequences of *S*. *halieti* (MN450344-MN450356) showed 98.6% (MN450340-MN450341) and 98.7% (MN450342-MN450343) identity with other sequences obtained in the present study; sequences N450340-MN450341 differed in three SNPs from MN450342-MN450343.

Based on ITS1 sequences, *S*. *columbae* was identified in one out of nine infected birds. Two other species, *S*. *lari* and *S*. *wobeseri*, were confirmed in two and three herring gulls, respectively; whereas the most common species, *S*. *halieti*, was observed in five birds. It should be emphasized, that two different *Sarcocystis* species were discovered in *L*. *argentatus* (No. 9) and (No. 26). *Larus argentatus* (No. 9) harboured *S*. *columbae* and *S*. *wobeseri*, while *L*. *argentatus* (No. 26) had sarcocysts of *S*. *halieti* and *S*. *wobeseri* (Table 1).

**Table 1:** *Sarcocystis* species diversity in nine herring gulls from Lithuania based on molecular identification of four sarcocysts from each bird

Host ID number	*S. columbae*	*S. halieti*	*S. lari*	*S. wobeseri*
7	–	+ (4)	–	–
9	+ (2)	–	–	+ (2)
14	–	–	–	+ (4)
21	–	–	+ (4)	–
23	–	+ (4)	–	–
26	–	+ (1)	–	+ (3)
29	–	+ (4)	–	–
32	–	–	+ (4)	–
35	–	+ (4)	–	–

*Notes*: Figures in parentheses show the number of isolates

*Key*: –, not found; +, detected

The morphological analysis of sarcocysts isolated from herring gulls indicated that *S*. *columbae*, *S*. *halieti*, *S*. *lari* and *S*. *wobeseri* are indistinguishable based on the size of sarcocysts and bradyzoites, as well as the thickness of the sarcocyst wall (Table 2). For instance, *S*. *wobeseri* had the thickest sarcocysts wall and *S*. *columbae* was distinguished by the thinnest cyst wall. However, morphological parameters of the four *Sarcocystis* species overlapped and it was impossible to discriminate these parasites under LM.

**Table 2:** Morphological characteristics of *Sarcocystis* species from herring gulls

Species	Size of sarcocysts	Sarcocyst wall thickness	Size of bradyzoites
*S*. *columbae*	3270–7800 × 90–120 (5935 × 105; *n* = 2)	0.7–1.1 (0.9; *n* = 2)	6.3–8.1 × 1.2–2.4 (7.0 × 1.9; *n* = 20)
*S*. *lari*	2860–7520 × 52–80; (6060 × 66.7; *n* = 8)	0.7–1.4 (1.1; *n* = 5)	6.2–8.9 × 1.2–2.0 87.2 × 1.6; *n* = 50)
*S*. *halieti*	3960–7930 × 43–128 (6221 × 75.6; *n* = 17)	0.8–1.2 (1.0; *n* = 12)	5.5–9.0 × 1.5–2.4 (7.1 × 1.9; *n* = 100)
*S*. *wobeseri*	3450–6900 × 70–200 (5189 × 120; *n* = 9)	0.7–1.5 (1.2; *n* = 7)	5.5–8.6 × 1.4–2.2 (7.0 × 1.8; *n* =30)

*Note*: Data (in micrometres) are presented as the range followed by the mean and the number of measurements in parentheses

## Discussion

In the present study four *Sarcocystis* species, *S*. *columbae*, *S*. *halieti*, *S*. *lari* and *S*. *wobeseri*, were identified in *L*. *argentatus* from Lithuania. These species had thread-like sarcocysts with a smooth cyst wall and were indistinguishable from one another under LM. Previously two *Sarcocystis* species were recorded in gulls: *S*. *wobeseri* was detected in *L*. *argentatus* [19] and *S*. *lari* was described based on material from *L*. *marinus* [15]. To our knowledge, *S*. *columbae* and *S*. *halieti* are detected in gulls for the first time in our study. Sarcocysts of *Sarcocystis* sp. detected in the muscles of the California gull (*Larus californicus*) from Canada had a thin (0.8 μm) and smooth cyst wall [5]. In Kazakhstan, Pak & Eshtokina [23] discovered sarcocysts with a thin and smooth cyst wall and banana-shaped bradyzoites in the black-headed gull (*L. ridibundus*) and the common gull (*L. canus*). Thus, the morphology of sarcocysts observed in the gulls from Canada and Kazakhstan is quite similar to those recorded in the present study.

The results of the present study indicate that not only *S*. *falcatula*, *S*. *calchasi* and *S*. *wobeseri* [10–13] but also *S*. *columbae* and *S*. *halieti* could form sarcocysts in birds belonging to different orders. Sarcocysts of *S*. *columbae* have previously been detected in the woodpigeon *C*. *palumbus* (Columbiformes) and *S*. *halieti* has been detected in *P*. *carbo* (Suliformes) [11, 24]. *Haliaeetus albicilla* and the Eurasian sparrow hawk (*Accipiter nisus*) have been confirmed as definitive hosts for *S*. *halieti* [6, 25]. *Accipiter nisus* does not prey on adult great cormorants and mainly feeds on small passerines [26]. Consequently, the range of the intermediate hosts of *S*. *halieti* might be much wider, whereas *S*. *lari* has been identified only in gulls, in *L*. *marinus* and in *L*. *argentatus* so far. Hence, further studies are needed to reveal the intermediate host specificity of avian *Sarcocystis* species.

Sarcocystis species richness detected in *L. argentatus* in the present study is greater than that found in other bird species. *Anas platyrhynchos* serves as an intermediate host for three *Sarcocystis* species, *S*. *anasi*, *S*. *rileyi* and *S*. *wobeseri* [10, 15, 27–30]. According to current knowledge, other birds can be involved as intermediate hosts for one or two *Sarcocystis* species [14–16, 31, 32]. The richness of *Sarcocystis* species observed in *L*. *argentatus* can be related to the wide geographical distribution and great variety of feeding habitats of this bird species, where herring gulls might ingest sporocysts shed by the definitive hosts [33, 34]. It should be noted, that breeding colonies of *L*. *argentatus* are often located in the areas that are also used by other gull species, cormorants and ducks, acting as intermediate hosts of *S*. *lari*, *S*. *halieti* and *S*. *wobeseri*, respectively [35].

The morphology of the sarcocysts wall is the main diagnostic feature for morphological separation of *Sarcocystis* species in intermediate hosts [1]. Under LM, a thin and smooth sarcocyst wall was described for several avian *Sarcocystis* species, *S*. *calchasi*, *S*. *columbae*, *S*. *corvusi*, *S*. *halieti*, *S*. *fulicae*, *S*. *lari* and *S*. *wobeseri*. These species also share similar sarcocyst wall structure under the transmission electron microscope [10, 11, 15, 16, 36, 37]. Thus, *Sarcocystis* species discussed are apparently morphologically indistinguishable. To the best of our knowledge, our study provides first evidence for several *Sarcocystis* spp. with a very similar morphological appearance under LM using a single bird species as an intermediate host. We have also demonstrated that one bird might host two *Sarcocystis* species, which could not be distinguished under LM. It should be emphasized, that the conclusions about *Sarcocystis* spp. richness in certain bird species might be misleading if only one sarcocyst is isolated for molecular identification. In 2011, our research group detected sarcocysts in the neck and leg muscles of four out of 11 herring gulls examined [19]. Under LM, one morphological type of sarcocyst was observed and only one excised cyst was subjected to molecular examination. At that time, it was assumed that sarcocysts detected in four herring gulls belonged to *S*. *wobeseri*. In contrast, the present study showed that *L*. *argentatus* can act as an intermediate host for four *Sarcocystis* species. Hence, when seeking to determine *Sarcocystis* species richness in birds, several sarcocysts should be isolated from each infected individual.

## Conclusions

In the present study four *Sarcocystis* species, *S*. *columbae*, *S*. *halieti*, *S*. *lari* and *S*. *wobeseri* were identified in *L*. *argentatus* from Lithuania by means of ITS1 sequence analysis. Detected *Sarcocystis* species were morphologically indistinguishable under LM. In comparison with other bird species, *L*. *argentatus* has the highest *Sarcocystis* species richness. The results of the present study showed that *S*. *columbae* and *S*. *halieti* could use birds of different orders as intermediate hosts. It was revealed that muscles of a single herring gull could be infected with two *Sarcocystis* species indistinguishable under LM; therefore, in order to determine *Sarcocystis* species richness in bird intermediate hosts, or at least within genus *Larus*, we recommend molecular characterization of several sarcocysts isolated from each infected individual.

## Data Availability

Data supporting the conclusions of this article are included within the article. The ITS1 sequences generated in the present study were submitted to the GenBank database under the accession numbers MN450338-MN450373.

## Abbreviations

LM: light microscopy
ITS1: internal transcribed spacer
SNP: single nucleotide polymorphism
IH: intermediate host
DH: definitive host
UV: ultraviolet
